# Feasibility of Ambient Sensing Technology for Fall Detection in People with Cognitive Changes

**DOI:** 10.1002/alz70858_101697

**Published:** 2025-12-25

**Authors:** Steven Ferguson, Shadi Gholizadeh, Timothy Thomas, Joey Taylor, Derek Gordon, Nathanial Findlay

**Affiliations:** ^1^ Lifeguard Health Inc., Québec, QC, Canada; ^2^ TheKey Research Group, San Diego, CA, USA; ^3^ TheKey, Irvine, CA, USA; ^4^ TheKey, Montreal, QC, Canada

## Abstract

**Background:**

Falls remain a leading cause of injury among individuals with cognitive changes, necessitating reliable detection and response systems. Ambient sensing technology provides 24/7 fall detection capabilities and customizable escalation protocols, aiming to minimize unnecessary calls to emergency dispatch services (911). This study explored the feasibility of implementing escalation processes to manage falls effectively within home care settings.

**Method:**

A feasibility study was conducted with 25 participants across 23 homes that had care for at least one month. Client care managers (*N* = 8) provided feedback from five cities: Montreal, Toronto, Winnipeg, Calgary, and Vancouver. Two modes of ambient sensing technology were utilized: one focused on falls and the other on broader monitoring capabilities, including wandering and inactivity detection. Both systems sent notifications to an alert center.

The alert center's process included:

1. Identifying the location and duration of the fall.

2. Checking the home to determine if the individual was alone.

3. Escalating to the “circle of friends,” a pre‐identified contact group, via phone or SMS.

4. If no response was received, the system could escalate to a 911 wellness check or client care manager, based on the families’ preferences.

Qualitative feedback was collected from families and care team staff to assess preferences and barriers to implementing the fall detection protocols.

**Result:**

Nearly all families (24 out of 25) indicated during assessment that they preferred escalation to the “circle of friends” rather than an automatic 911 dispatch, highlighting that emergency response is not always the desired solution. Participants appreciated the flexibility and customization of the escalation process. The inactivity alert system, which learns daily habits, successfully minimized false alarms by sending SMS notifications to confirm well‐being before escalating.

**Conclusion:**

Ambient sensing technology for fall detection is a feasible and acceptable solution when supported by tailored escalation protocols. The inclusion of a “circle of friends” approach ensures a personalized response, reducing reliance on 911 and addressing concerns about unnecessary interventions. Future research will further refine these protocols and validate their effectiveness in diverse care settings.

